# Simulation and Performance Analysis of Dielectric Modulated Dual Source Trench Gate TFET Biosensor

**DOI:** 10.1186/s11671-021-03486-2

**Published:** 2021-02-12

**Authors:** Chen Chong, Hongxia Liu, Shulong Wang, Shupeng Chen

**Affiliations:** Key Laboratory for Wide Band Gap Semiconductor Materials and Devices of Education, School of Microelectronics, Xidian University, Xi’an, 710071 China

**Keywords:** Dielectric modulated dual source trench gate tunnel FET (DM-DSTGTFET), Biosensor, Sensitivity

## Abstract

In this paper, a dielectric modulated double source trench gate tunnel FET (DM-DSTGTFET) based on biosensor is proposed for the detection of biomolecules. DM-DSTGTFET adopts double source and trench gate to enhance the on-state current and to generate bidirectional current. In the proposed structure, two cavities are etched over 1 nm gate oxide for biomolecules filling. A 2D simulation in the Technology Computer-Aided Design (TCAD) is adopted for the analysis of sensitivity study. The results show that under low supply voltage, the current sensitivity of the DM-DSTGTFET is as high as 1.38 × 10^5^, and the threshold voltage sensitivity can reach 1.2 V. Therefore, the DM-DSTGTFET biosensor has good application prospects due to its low power consumption and high sensitivity.

## Introduction

In the recent past, significant research interest has been focused on silicon-based field effect transistor (FET) biosensors owing to the promising characteristics of high sensitivity, minimum delay, scaled dimensions and low cost [1–6]. FET-based biosensors have the limitation of thermal electron emission and have a subthreshold slope (SS) that can be more than 60 mV/decade. Due to the band-to-band-tunneling (BTBT) conduction mechanism, the TFET overcomes the limitation and lowers the short channel effect [7–10]. Hence, TFET-based biosensor has emerged as a suitable candidate for better sensitivity and response time than FET-based biosensor [11–14].

The most common method in TFETs applied for molecule detection is based on dielectric modulation. A portion of the gate dielectric material is etched out to form a cavity; when biomolecules are filled in the cavity, the dielectric constant of the cavity changes, and a change is reflected in the drain current and transfer characteristics [15–17]. At the same time, dielectric modulation aids in sensing both charged and neutral molecules. Presently, the concept of dielectric modulation has been recently utilized in TFET, and the dielectrically modulated TFET (DMTFET)-based biosensor has attracted the highly valued of researchers. A p-n-p-n TFET working as a biosensor for label-free biomolecule detection is studied with device simulation. Results reveal that a TFET-based biosensor has low off-state current in the absence of biomolecules and high sensitivity toward both dielectric constant and charge[18].It has been observed in [19] that the presence of biomolecules in the cavity near the tunnel junction can lead to effective coupling, which leads to high sensitivity, and also makes the DM-TFET resistant to sensitivity reduction at a lower dimension. TFET-based biosensors of different structures are being studied. Compared with the traditional DGTFET, incorporating the short gate (SG) architecture into the DMTFET structure can significantly improve sensitivity and reduce cost [20]. Charge-plasma-based gate underlap dielectric modulated junctionless tunnel field-effect transistor (CPB DM-JLTFET) can obtain the maximum sensitivity (neutral and charged biomolecules) by appropriately selecting the length and thickness of the cavity near the tunnel junction under the appropriate bias [21]. To improve the sensitivity of the biosensor, a heavily doped front gate n + pocket and gate-to-source overlap is introduced in a vertical dielectrically modulated tunnel field-effect transistor (V-DMTFET) [22]. Circular gate Heterojunction tunnel field effect transistor exhibits higher sensitivity than uniform gate HJ TFET due to its non-uniform gate architecture [23]. Double channel trench gate TFET exhibits high current sensitivity as well as exorbitant voltage sensitivity [24]. The double gate and double metal materials TFET biosensor can make the sensitivity change more obvious [25].

However, most of the biosensors are based on the double-gate TFET, in which the biomolecules need to be added from the sides of the gates at both ends. In the proposed structure, the biomolecules are added vertically from the top of the device, which is a simpler operation. In addition, because the gate-source overlap area is large, that is, the area where the source and biomolecules interaction is obvious, the sensitivity of DM-DSTGTFET biosensor is higher than other devices, as shown in Table 1. Table 1 summarizes the comparison of the different sensitivities between this work and the research results in other references.

**Table 1:** Sensitivity comparison of DM-DSTGTFET biosensor with other reported TFET biosensors

Sensitivity parameter	Ref [12]	Ref [18]	Ref [21]	Ref [22]	Ref [23]	Ref [26]	Ref [27]	Ref [28]	This work
*I*_on_/*I*_off_	9 × 10^9^	1 × 10^8^	1.09 × 10^10^	1 × 10^9^	1 × 10^6^	1.2 × 10^10^	-	1 × 10^7^	1.1 × 10^10^
△*V*_th_	0.7	–	–	–	–	–	0.45	–	1.2
*S*_drain_	–	–	1934	300	1 × 10^5^	100	–	–	1.38 × 10^5^
S_SS_	0.5	–	–	0.5	–	–	–	–	0.8

In this paper, the sensitivity of DM-DSTGTFET biosensor is studied and the specific content is as follows. Sections 2 and 3 describe basic device structure, fabrication process, simulation model and method. Section 4 characterizes the effect of different factors on the sensitivity of DM-DSTGTFET biosensor. Specifically, the influences of different dielectric constants, cavity thickness and charged biomolecules on Transfer characteristics, the *I*_on_/*I*_off_ sensitivity and △*V*_th_ sensitivity of proposed device. Section 5 concludes the research findings from the investigation performed.

## Device Structures

**Fig.1:**
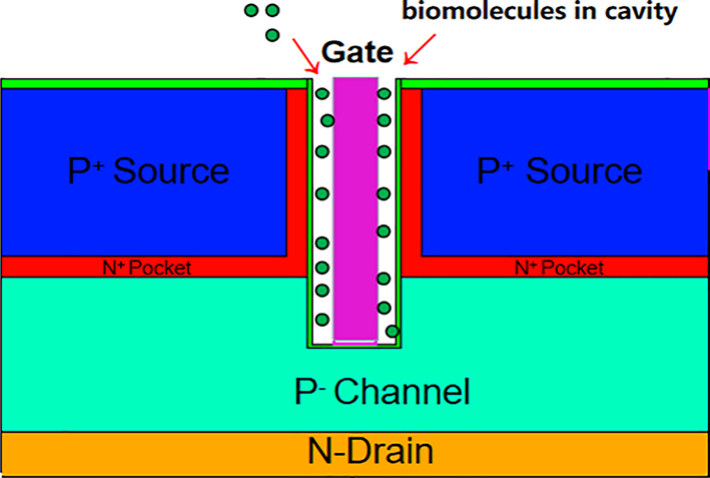
Schematic cross-sectional view of DM-DSTGTFET biosensor

Figure 1 shows a cross-sectional image of DM-DSTGTFET-based biosensor. The gate electrode of DM-DSTGTFET has a work function of 4.2. In order increasing the on-state current of the TFET, a dual source structure is utilized. The two source regions with doping concentration of 1 × 10^20^ cm^−3^ are placed symmetrically on both sides of the gate. The p-channel with height (H_c_) of 37 nm and doping concentration of 1 × 10^15^ cm^−3^ is below the source and gate. The n-drain with doping concentration of 1 × 10^17^ cm^−3^ and height (*H*_d_) of 18 nm is below the channel. Two oxides on the source regions are HfO_2_ with a thickness of 2 nm. The two pocket regions of thickness (*T*_p_) 5 nm are symmetrically placed on either side of the gate with donor doping concentration of 1 × 10^19^ cm^−3^. Additionally, for the proposed biosensor, *T*_ox_ (1 nm), *T*_c_ (5 nm) are the thickness of the HfO_2_ gate oxide and width of nanogap cavity, respectively. To facilitate an appropriate change in the sensitivity parameter, the value of gate metal work function chosen should be such that tunneling could occur only whenever the biomolecules accumulated in the cavity. That is why metal work function Φ_MS_ = 4.2 eV (over the HfO_2_ gate oxide) is chosen. Now, five different types of small biomolecules with different dielectric constants (1, 2.5, 5, 11, 23) and five different thickness of nanogap cavity (5 nm, 7 nm, 9 nm, 11 nm, 13 nm) is analyzed for the proposed biosensor.

**Fig.2:**
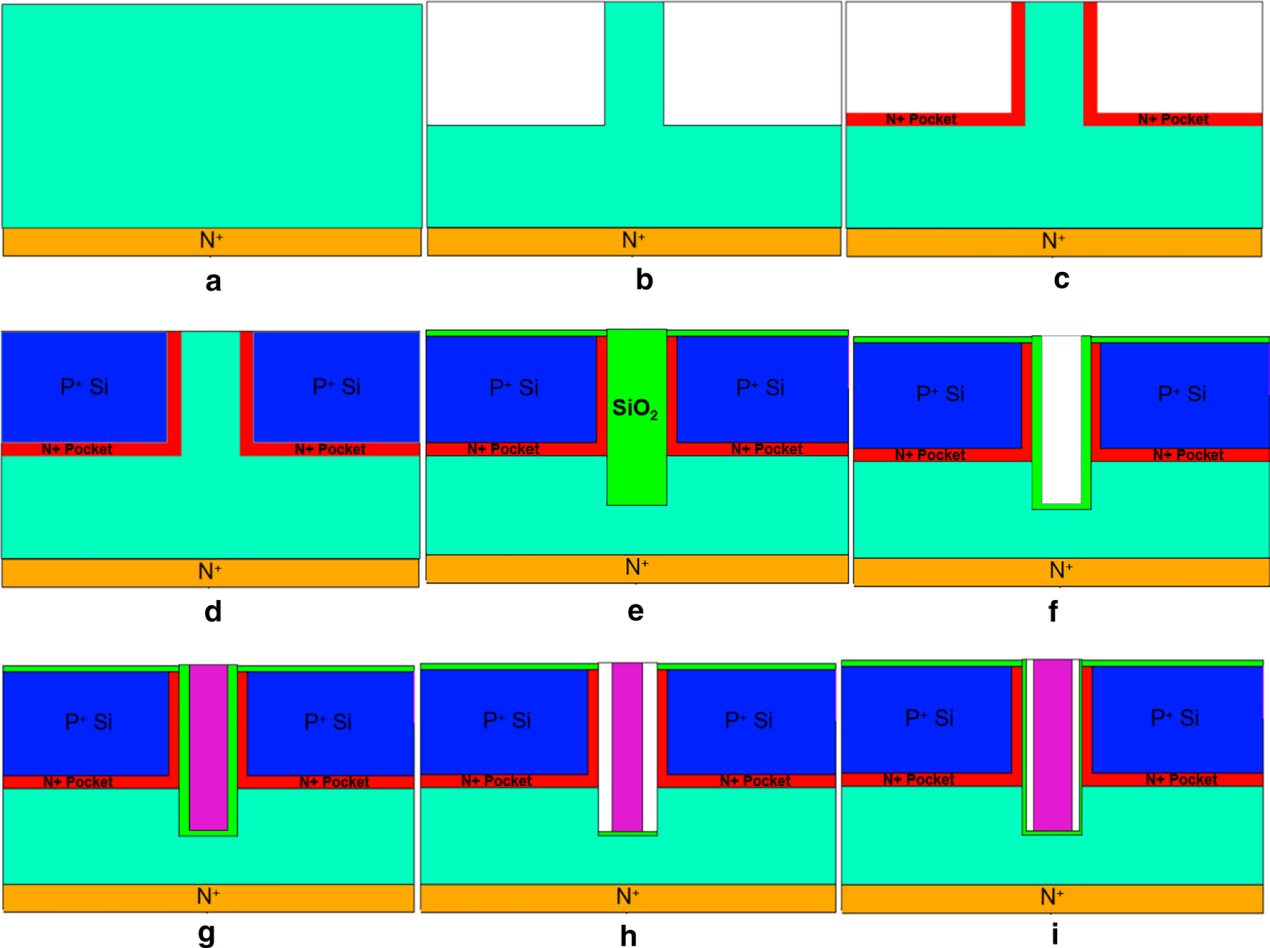
Fabrication flow for DM-DSTGTFET as biosensor

The fabrication method of the DM-DSTGTFET is similar with the published [24]. Figure 2 shows the fabrication steps of the proposed DM-DSTGTFET. In the first step, as shown in Fig. 2a, through a mask, exposure, etching, ion implantation, and annealing on a lightly doped silicon substrate, a drain region at the bottom of the device is formed. The doping concentration of the formed drain region is 10^17^/cm^3^, and the doping ion is arsenic. Then intrinsic silicon is epitaxially grown on top of the drain region to form the channel region of the device. As shown in Fig. 2b, the two corners above the channel are etched away. Simultaneously, N^+^ doping is deposited by chemical vapor deposition (CVD) technique as described in Fig. 2c to form the pocket regions of DM-DSTGTFET. In the source region, a Si-based dual source region is grown by chemical vapor deposition (CVD), and masking, exposure, etching, ion implantation, and annealing are performed for P-type highly doping in the source region, with a doping concentration of 10^20^/cm^3^, as shown in Fig. 2d. In the next step, the trench is made in channel layer and SiO_2_ is deposited in the trench as given in Fig. 2e. Then trench is formed as depicted in Fig. 2f. The metallization and patterning are carried out to obtain the gate contacts as shown in Fig. 2g. Further, the cavities are carved in SiO_2_ on both sides of the gate as given in Fig. 2h. In the final step, 1 nm HfO_2_ is grown on the side wall of cavities to obtain the proposed structure as depicted in Fig. 2i.

## Simulation Method and Model

For the purpose of studying the performance of DM-DSTGTFET biosensors more clearly, this paper utilizes TCAD tool (sentaurus) to study the sensitivity of TFET sensors. The appropriate models are adopted for accurate simulation.

The nonlocal BTBT model considers the electric field at each point in the tunneling path as a variable, which means the BTBT tunneling probability depends on the band bending at the tunneling junction. The non-local tunneling model is more in line with the actual situation of TFET simulation [29]. Hence, the nonlocal BTBT model is adopted in this paper. The Kane model is used for the dynamic non-local BTBT tunneling model in sentaurus. In the Kane model, the rate of BTBT tunneling is expressed as [30]:

1 $$G_{{{\text{BTBT}}}} = A\left( {\frac{E}{{E_{0} }}} \right)^{P} \exp \left( { - \frac{B}{{E_{0} }}} \right)$$

where constant *E*_0_ = 1 V/cm, P = 2 for direct band gap tunneling, and *P* = 2.5 for phonon-assisted indirect band gap tunneling. Since the devices in this paper are mainly silicon, *P* choose 2.5. The parameter *A* = 4 × 10^14^/cm^3^s, *E* is the electric field and the exponential factor *B* = 9.9 × 10^6^ V/cm.

Shockley–Read–Hall (SRH) is chosen to include the recombination of carriers. Band gap narrowing model is taken to activate the high concentration effect in the band gap. Fermi–Dirac statistics is invoked to include the change in properties of a highly doped region. The mobility model in Si material should consider the scattering model of ionized impurities (*µ*_dop_), the interface scattering model (*µ*_InterSc_) and the high-field saturation model (*µ*_F_) [31], and the final effective mobility model can be expressed by:

2 $$\frac{{1}}{\mu } = \frac{{1}}{{\mu_{{{\text{dop}}}} }} + \frac{1}{{\mu_{{{\text{InterSc}}}} }} + \frac{1}{{\mu_{{\text{F}}} }}$$

Poole–Frenkel mobility model is introduced in the material filling the cavity, and the mobility as a function of the electric field is given by:

3 $$\mu = \mu_{{0}} \exp \left( { - \frac{{E_{0} }}{KT}} \right)\exp \left( {\sqrt E \left( {\frac{\beta }{T} - \gamma } \right)} \right)$$

where *µ*_0_ is the low-field mobility, *β* and *γ* are fitting parameters, *E*_0_ is the effective activation energy, and E is the driving force (electric field). *K* is the Boltzmann constant, and *T* is the temperature. The default value of *E*_0_ and γ is 0, β = 0.1.

Based on the above calibrated physical model, the electrical characteristics of DM-DSTGTFET biosensor are analyzed.

During simulation, four different dielectric constants biomolecules (*k* = 2.5, 5, 11, 23), five cavity thickness (*T*_c_ = 5, 7, 9, 11, 13 nm) and different densities of charged biomolecules are considered in simulation and discussion. In general, a reference is adopted when studying the sensitivity of the sensor. The reference is proposed which can make the sensor's response to the target substance obvious. Hence, the reference is taken in the case when the cavities are filled with air, or simply, the condition when the biomolecules are not filled in the cavities. Therefore, a measure of threshold voltage sensitivity, drain current sensitivity and subthreshold slope sensitivity of the DM-DSTGTFET is defined as [22] [28] [32]:

4 $$\Delta V_{{{\text{th}}}} = V_{{\text{th(air)}}} - V_{{\text{th(bio)}}}$$

5 $$S_{{{\text{drain}}}} = \frac{{I_{{\text{ds(bio)}}} - I_{{\text{ds(air)}}} }}{{I_{{\text{ds(air)}}} }}$$

6 $$S_{{{\text{SS}}}} = \frac{{SS_{{{\text{air}}}} - SS_{{{\text{bio}}}} }}{{SS_{{{\text{air}}}} }}$$

where *V*_th(air)_ is the threshold voltage of the biosensor when the cavities are filled with air, and *V*_th(bio)_ is the threshold voltage when the cavities are filled with biomolecules. Similarly, *I*_ds(air)_ and SS_air_ are the on-state drain current and subthreshold swing, respectively, of the biosensor when the cavities are filled with air, and *I*_ds(bio)_ and SS_bio_ are the on-state drain current and subthreshold swing, respectively, when the cavities are filled with biomolecules.

Through the analysis of the electrical characteristics of the DM-DSTGTFET, the threshold voltage, on-state drain current and subthreshold swing are extracted to analyze the sensitivity of the biosensor.

## Results and Discussion

### Impact of Different Biomolecules in DM-DSTGTFET

**Fig.3:**
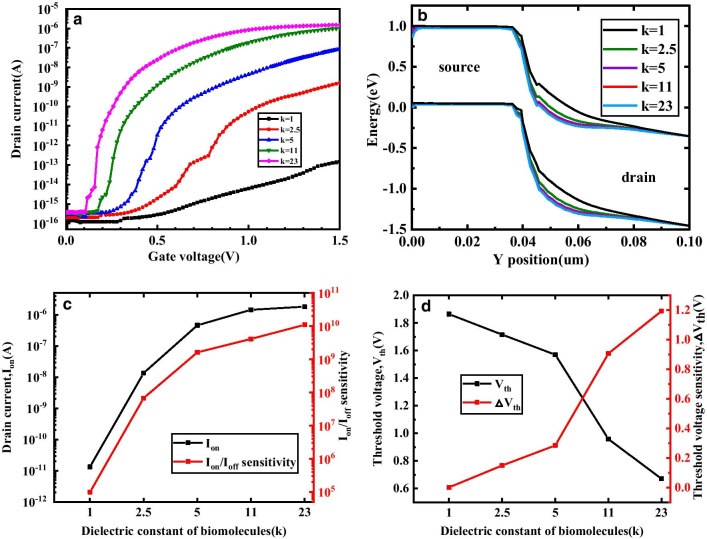
**a **Transfer characteristics, **b** energy bands variation with respect to the y axis, **c** *I*_on_/*I*_off_ sensitivity and **d** threshold voltage sensitivity of DM-DSTGTFET biosensor for different values of k at V_d_ = 0.5 V and *T*_c_  = 5 nm

Figure 3 shows the transfer characteristic, energy band variation, threshold voltage sensitivity and current sensitivity of DM-DSTGTFET in the on-state when different dielectric constant of biomolecules fill the cavity. By choosing a lower gate metal work function (Φ_MS_ = 4.2), the sensitivity of the drain current can be studied by adjusting the different k.

**Fig.4:**
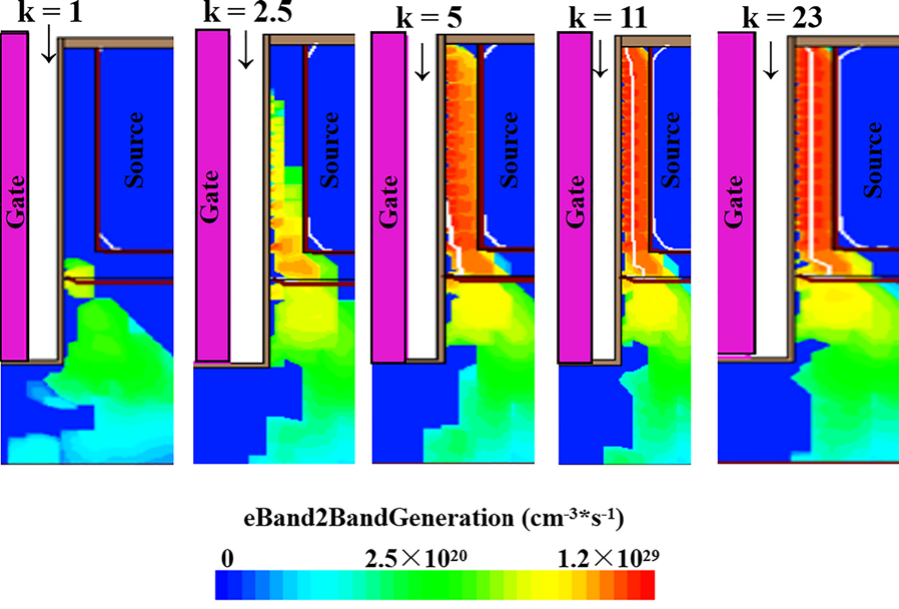
Electron BTBT generation in DM-DSTGTFET biosensor for different biomolecules when Vd = 0.5 V, *T*_c_ = 5 nm and Vg = 1.5 V

As can be seen in Fig. 3a, with the k of the gate dielectric increases, the stronger the gate control capability, the on-state current also increases. Figure 3b describes the energy band diagram at the different k of biomolecules. When *k* = 1, it means there is no biomolecules filled in the cavity. In this case, twist in the energy band is minimized. Moreover, when the dielectric of the biomolecules constant in the cavity starts increasing, the energy band bends more and more severely. It means more energy band alignment takes place at higher *k*, and thus barrier width across the junction reduces. Figure 3c shows the effect of dielectric constant of biomolecules on *I*_on_ and *I*_on_/*I*_off_ sensitivity of DM-DSTGTFET. With the increase in *k*, the *I*_on_ and *I*_on_/*I*_off_ sensitivity also improves. This is because of a fact that with increase in k, the more severe the energy band bending, the barrier width at the source-channel junction is decreased and hence the tunnel possibility increases. As the tunneling probability increases, the electron BTBT tunneling generation increases which can be seen clearly in Fig. 4. The proposed device provides the highest *I*_on_/*I*_off_ sensitivity of 1.1 × 10^10^ at *k* = 23, which is obviously higher than the published TFET-based biosensors. Figure 3d gives the variation in *V*_th_ and △*V*_th_ sensitivity of DM-DSTGTFET with respect to the k of biomolecules. Obviously, as the *k* increases, the faster the *I*_on_ of the proposed device increases, the lower the threshold voltage. Meanwhile, the △*V*_th_ shows an increasing trend with rise in *k*. The reason is that the difference between the *V*_th_ when different biomolecules are filled and the *V*_th_ when no biomolecule is filled is getting larger. In general, the *V*_th_ when filled with air is larger than other k values. The proposed DM-DSTGTFET performs a maximum △*V*_th_ sensitivity of 1.2 V at *k* = 23. Therefore, the DM-DSTGTFET provides high current sensitivity as well as threshold voltage sensitivity for biomolecules.

**Fig.5:**
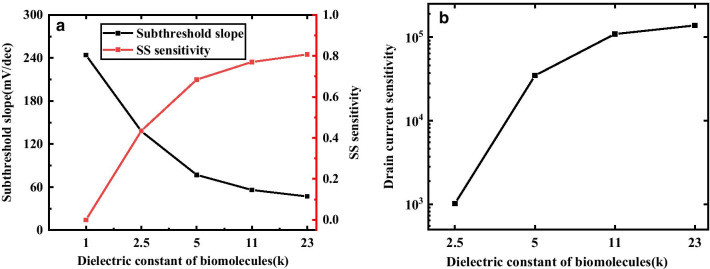
**a** Subthreshold slope, subthreshold slope sensitivity and **b** drain current sensitivity with different biomolecules when V_d_ = 0.5 V, *T*_c_ = 5 nm and V_g_ = 1.5 V

Figure 5a shows SS and SS sensitivity of DM-DSTGTFET when the cavities are filled with different biomolecules. Here, it is seen that the increment in dielectric constant, results in decrease of SS and improvement of S_SS_. The smaller the SS, the smaller the power consumption of the TFET, and the better the performance of the TFET. Therefore, As the value of k increases, SS decreases, S_SS_ increases, and gate control capability increases.

The drain current sensitivity varies as a function of k for the proposed DM-DSTGTFET in Fig. 5b. The sensitivity increases with increase in k. This is due the fact that increase in k results in enhancement of electric field at the tunnel junction which leads to reduction in the tunnel width and hence increases *S*_drain._

### Impact of Different Cavity Thickness in DM-DSTGTFET

Because when *k* = 23, the *S*_drain_, △*V*_th_ sensitivity and S_SS_ of the DM-DSTGTFET biosensor are the largest (the conclusion drawn from the previous section). Therefore, in order study the influence of the cavity thickness on the sensitivity of the proposed biosensor more clearly, this section is conducted under the condition of *k* = 23.

**Fig.6:**
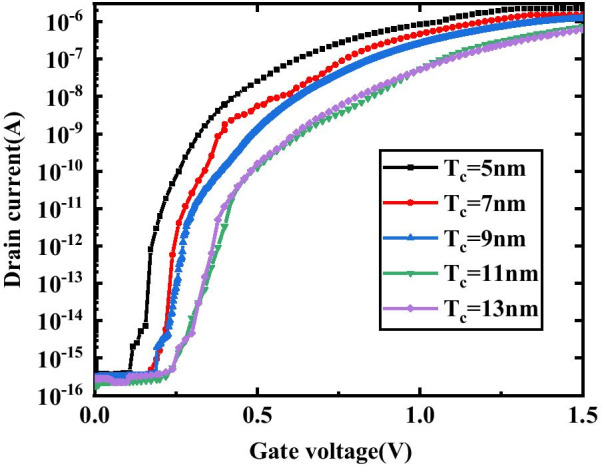
Transfer characteristics of DM-DSTGTFET biosensor for different values of cavity thickness (*T*_c_) at V_d_ = 0.5 V, V_g_ = 1.5 V and *k* = 23

**Fig.7:**
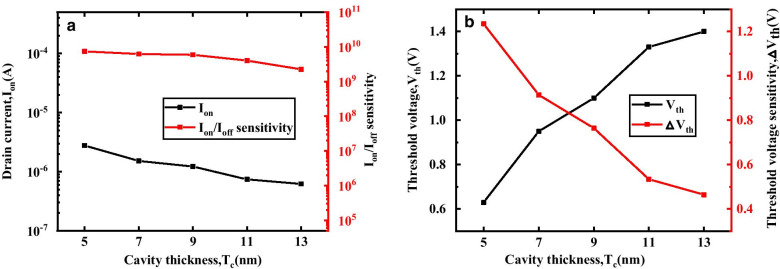
**a** Effect of different values of cavity thickness (*T*_c_) on* I*_on_, *I*_on_/*I*_off_ sensitivity, **b** *V*_th_ and △*V*_th_ of DM-DSTGTFET at V_g_ = 1.5 V, V_d_ = 0.5 V and *k* = 23

Figure 6 describes the transfer characteristics of the DM-DSTGTFET biosensor at different cavity thicknesses (*T*_c_). As *T*_c_ increases, the on-state current becomes smaller. The effect of different *T*_c_ on *I*_on_ and *I*_on_/*I*_off_ sensitivity of DM-DSTGTFET is plotted in Fig. 7a. When *T*_c_ is increased, the capacitance between gate and channel is reduced which leads to larger tunnel width at the source-channel junction resulting in lower drain current. For *k* = 23, the *I*_on_ and *I*_on_/*I*_off_ sensitivity decreases with increase in *T*_c_ due to improvement in capacitive coupling between gate and channel for higher *T*_c_. On the other hand, the proposed device exhibits an increasing trend in *V*_th_ and hence in △*V*_th_ sensitivity with increase in *T*_c_ as illustrated in Fig. 7b. This is because the increase in *T*_c_ reduces the *I*_on_ and hence increases the *V*_th_. In other words, the control of gate over channel reduces for wider cavity which leads to higher *V*_th_. Therefore, the DM-DSTGTFET works as a better voltage biosensor for a narrower cavity.

### Impact of Charged Biomolecules on DM-DSTGTFET

To investigate the influence of the different charges of biomolecules on the sensitivity of the proposed sensor, the dynamic range and detection limit were first studied. In this paper, the DM-DSTGTFET can detect the sensing material with a charge density ranging from 10^10^ cm^−2^ to 10^13^ cm^−2^, a wider detection range compared to other sensors [32]. Therefore, in the following simulation, the charge density within the dynamic limit range is used for sensitivity research.

**Fig.8:**
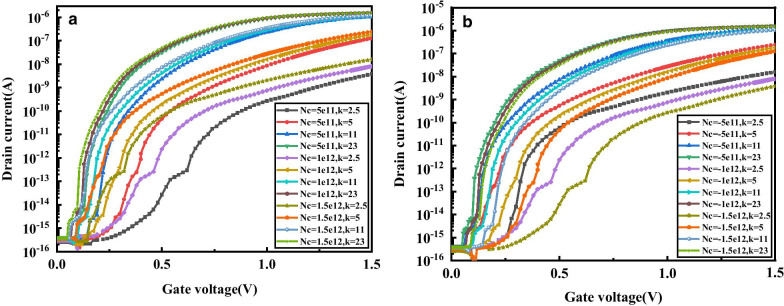
Transfer characteristics of DM-DSTGTFET biosensor for dielectric constant of biomolecules, **a** different positive charge and b different negative charge of biomolecules at V_d_ = 0.5 V, V_g_ = 1.5 V and *T*_c_ = 5 nm

Figure 8 depicts the effect of filling the cavity with biomolecules with different positive charges and negative charges on the transfer characteristics of the DM-DSTGTFET under different *k*. As can be seen, at *k* = 2.5, under biomolecules positively and negatively charged, the transfer curve has a larger change range. Therefore, the following discussion focuses on the effect of different positive charges and negative charges on the sensitivity of DM-DSTGTFET biosensor when *k* = 2.5.

**Fig.9:**
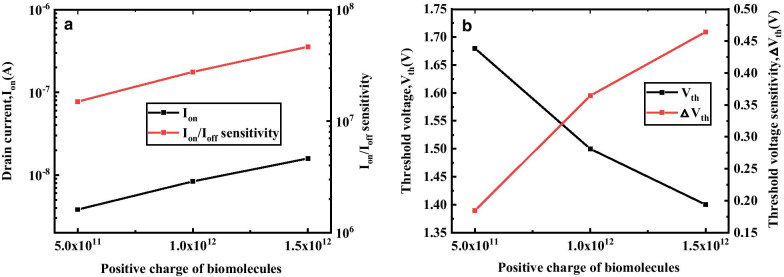
**a** Effect of different positive charge of biomolecules on* I*_on_, *I*_on_/* I*_off_ sensitivity, **b** *V*_th_ and △*V*_th_ of DM-DSTGTFET at V_g_ = 1.5 V, V_d_ = 0.5 V, *k* = 2.5 and *T*_c_ = 5 nm

Figure 9a describes the variation of Ion and *I*_on_/*I*_off_ sensitivity of DM-DSTGTFET as a function of positive charges. The increasing positive charge of biomolecules leads to improvement in Ion and *I*_on_/*I*_off_ sensitivity of the proposed device. The positive charge in the cavity increases the effective gate oxide dielectric which results in enhancement of gate control ability. This increase in gate control ability causes decrease in tunneling width of source-channel junction leading to improvement in *I*_on_ and *I*_on_/*I*_off_ sensitivity. Figure 9b demonstrates the effect of positive charge of biomolecules on *V*_th_ and △*V*_th_ sensitivity of the DM-DSTGTFET. It is observed that the *V*_th_ reduces and △*V*_th_ sensitivity improves with increase in positive charge. This is due the fact that the positive charge on the molecule increase the *I*_on_ and decreases *V*_th_. The decrease in *V*_th_ enhances the difference between the threshold voltage of biomolecule with respect to air leading to improvement in △*V*_th_.

**Fig.10:**
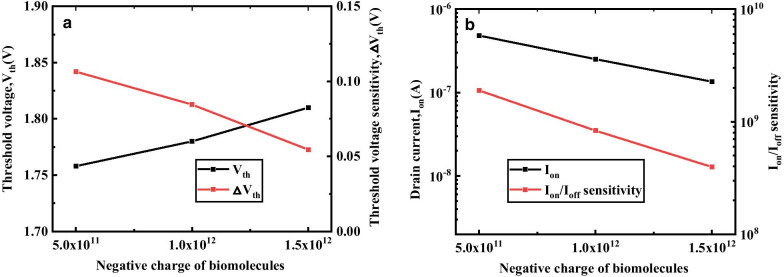
**a** Effect of different negative charge of biomolecules on *I*_on_, *I*_on_/* I*_off_ sensitivity, **b** *V*_th_ and △*V*_th_ of DM-DSTGTFET at V_g_ = 1.5 V, V_d_ = 0.5 V, *k* = 2.5 and *T*_c_ = 5 nm

Figure 10a shows the variation of *I*_on_ and *I*_on_/*I*_off_ sensitivity of DM-DSTGTFET as a function of positive charges for *k* = 2.5. The increasing negative charge of biomolecules leads to decrease in Ion and *I*_on_/* I*_off_ sensitivity of the proposed device. The negative charge in the cavity decreases the effective gate oxide dielectric which results in enhancement of gate control ability. This decrease in gate control ability causes increase in tunnel width of source-channel junction leading to decrease in Ion and *I*_on_/*I*_off_ sensitivity.

Figure 10b demonstrates the effect of negative charge of biomolecules on *V*_th_ and △*V*_th_ sensitivity of the DM-DSTGTFET. It is observed from the figure that for *k* = 2.5, the *V*_th_ improves and △*V*_th_ sensitivity reduces with increase in negative charge. This is due the fact that the negative charge on the molecule decreases the *I*_on_ and increase *V*_th_. The increase in *V*_th_ enhances the difference between the threshold voltage of biomolecule with respect to air leading to decrease in △*V*_th_.

## Conclusions

In conclusion, DM-DSTGTFET has high sensitivity for detection of biomolecules in biosensor applications. However, the detecting ability of DM-DSTGTFET structure is evaluated by examining the effects introduced by relative permittivity, cavity thickness, charged biomolecules, *I*_on_/*I*_off_ sensitivity, SS and S_SS_. The results show that the larger the dielectric constant, the smaller the thickness of the cavity, the more positively charged, and the greater the sensitivity of the proposed device. Simulation results show that the proposed structure can be applied for ultra-sensitive and low-consumption biosensor device.

## Abbreviations

DM-DSTGTFETS: Dielectric modulated dual source trench gate tunnel field-effect transistors
TCAD: Technology computer-aided design
BTBT: Band-to-band-tunneling
DGTFET: Dual gate tunnel field-effect transistors
SS: Subthreshold slope
